# Improved robustness of sequentially deposited potassium cesium antimonide photocathodes achieved by increasing the potassium content towards theoretical stoichiometry

**DOI:** 10.1038/s41598-025-87603-6

**Published:** 2025-01-23

**Authors:** Lei Guo, Keisuke Shiohara, Hisato Yamaguchi, Gaoxue Wang, Yuki Okabe, Masashi Nakatake, Shoichi Takakura, Masahiro Yamamoto, Shuichi Ogawa, Yoshifumi Takashima

**Affiliations:** 1 Nagoya University, Furo, Chikusa, Nagoya, Aichi 464-8601 Japan; 2 Los Alamos National Laboratory, P.O. Box 1663, Los Alamos, NM 87545 USA; 3 Nihon University, 1-2-1 Izumi-Cho, Narashino-Shi, Chiba, 275-8575 Japan; 4 Aichi Synchrotron Radiation Center, Aichi Science & Technology Foundation, Seto, Aichi 489-0985 Japan; 5 High Energy Accelerator Research Organization, 1-1 Oho, Tsukuba, Ibaraki 305-0801 Japan

**Keywords:** Materials science, Nanoscience and technology

## Abstract

Alkali antimonide semiconductor photocathodes are promising candidates for high-brightness electron sources for advanced accelerators, including free-electron lasers (FEL), due to their high quantum efficiency (QE), low emittance, and high temporal resolution. Two challenges with these photocathodes are (1) the lack of a universal deposition recipe to achieve crystal stoichiometries and (2) their high susceptibility to vacuum contamination, which restricts their operation pressure to ultrahigh vacuums and leads to a short lifetime and low extraction charge. To resolve these issues, it is essential to understand the elemental compositions of deposited photocathodes and correlate them to robustness. Here, we report depth profiles for potassium cesium antimonide photocathodes, which were investigated using synchrotron radiation x-ray photoelectron spectroscopy, and the robustness of those photocathodes. We prepared two types of photocathodes with different potassium contents via sequential thermal evaporation. Depth profiles revealed that the photocathodes with a potassium deficit had excess cesium at the surface, while the ratio of potassium and cesium to antimony decreased rapidly within the film. In contrast, the photocathodes with sufficient potassium had close to the theoretical stoichiometry of K_2_CsSb at the surface and maintained that stoichiometry for over half the entire film thickness. Both photocathode types had a similar maximum QE at 532 nm; however, exposure to oxygen revealed that the photocathode with a crystalline stoichiometry of K_2_CsSb maintained QE at one order of magnitude higher pressure compared to its potassium-deficit counterpart. These results highlight the importance of synthesizing potassium cesium antimonide photocathodes with sufficient potassium to achieve the theoretical crystalline stoichiometry for both high QE and improved robustness.

## Introduction

High-brightness coherent light offers a unique opportunity to capture a phenomenon that no other method is capable of capturing, such as molecular dynamics in real time (i.e., femtoseconds)^1–3^. A particle accelerator, specifically a free-electron laser (FEL) linear particle accelerator (linac), is required to generate such light^4,5^. The factors essential to achieving high beam brightness are a large peak current, a low emittance, a short pulse time, and a high repetition rate, where emittance is defined as the volume of the six-dimensional phase space of the position and momentum of the beam. A short pulse time and high repetition rate can be readily attained by using photocathodes instead of thermionic cathodes. Therefore, photocathodes have been used to operate advanced accelerators since the 1980s, including the Linac Coherent Light Source (LCLS) of the Stanford Linear Accelerator Center (SLAC)^6^. Because of their reliability and robustness, metals such as copper (Cu), magnesium (Mg), and silver (Ag) are the most common type of photocathodes used to generate an FEL in linear accelerator facility operations^7–9^. The drawback of these metals is large work functions, which require ultraviolet (UV) light for photo-electron emission and result in a low quantum efficiency (QE) of approximately 0.01%. Third or fourth harmonics of a short-pulse infrared (IR) or visible-light (VL) laser are generally required to achieve the necessary UV light; thus, optical setups are complex, resulting in low conversion efficiency and low laser stability^10^. QE is defined as the number of photoelectrons emitted per incident photon, thus, a low QE leads to a low peak current. Alternatives to metal photocathodes include cesiated gallium arsenide (GaAs), which exhibits a high QE of up to 10% with VL excitation^11^. This high QE has led to the use of cesiated GaAs in the compact energy recovery linac (cERL) at the High Energy Accelerator Research Organization in Japan^12,13^. The cERL beam requirement is more than 10 mA average current, less than 1 mm·mrad normalized emittance, and a long photocathode lifetime of several weeks or more (equivalent to 10^4^ C or more as the amount of extraction charge). The challenge is the extreme susceptibility of cesiated GaAs to environmental conditions because of an atomically thin layer of an alkali metal, cesium, that is used to create negative electron affinity surfaces (operates at 10^–9^ Pa)^14^. Cesium telluride (Cs_2_Te) photocathodes also exhibits a high QE of more than 10% and are more robust than cesiated GaAs (operates at 10^–7^ to 10^–6^ Pa). This makes Cs_2_Te photocathodes one of the attractive candidates for generating a high-brightness electron beam, which is why these photocathodes are being used in the last-generation x-ray FEL (XFEL)-based user facility^15–17^. However, Cs_2_Te photocathodes require UV excitation similar to the metal photocathode.

Alkali antimonide photocathodes such as cesium potassium antimonide (K_2_CsSb), have well-balanced properties; thus, they are attracting attention as next-generation photocathodes. Specifically, K_2_CsSb has a QE that exceeds 5% at 532 nm excitation, less than 0.4 µm·rms normalized emittance, and can operate at 10^–8^ Pa, which is one order of magnitude higher than the pressure at which negative-electron-affinity GaAs photocathodes can operate^18–29^. A record-high beam current of 60 mA in a DC injector with a 30 h 1/e lifetime has been demonstrated^19^. Based on these characteristics, K_2_CsSb is a candidate photocathode material for LCLS II at SLAC, which has a required performance of current up to 0.3 mA and 0.4 µm·rms normalized emittance to achieve the first continuous-wave XFEL^30,31^. However, the pressing technical challenges for implementing K_2_CsSb photocathodes on accelerators are (1) the lack of a universal deposition recipe to achieve crystal stoichiometry and (2) its susceptibility to vacuum contamination. To tackle these challenges, the first step is to understand the correlation between the deposition recipe and the elemental compositions of photocathode thin films, especially regarding depth. The next step is to correlate elemental compositions with robustness.

In this study, we used a vacuum suitcase to transport K_2_CsSb photocathodes to a synchrotron radiation facility beamline where we performed elemental composition analysis. Specifically, we sequentially deposited K_2_CsSb photocathodes with two different potassium contents and compared their depth profiles. Elemental compositions were quantitatively analyzed with x-ray photoelectron spectroscopy and ion sputtered to obtain depth profiles. QE at 532 nm under different pressures of oxygen gas was also compared. Our results provide insight into the importance of establishing a deposition recipe to achieve the stoichiometry of crystalline K_2_CsSb to maximize both QE and robustness.

## Results and discussion

We routinely observed QE of over 5% at 532 nm with our deposition recipe for potassium cesium antimonide thin films^24,26^. This QE is comparable to the high QE reported for potassium cesium antimonide; thus, our assumption was that our films possess the stoichiometry of crystalline K_2_CsSb. However, because of the extreme susceptibility of films, which start to degrade at 10^–7^ Pa, we had not confirmed the stoichiometry. To characterize the films without degradation, we designed and fabricated vacuum suitcases capable of maintaining a pressure of 10^–8^ Pa and used them to transport the samples from our deposition chamber to a synchrotron facility for measurements using high-brightness x-ray.

**Fig. 1:**
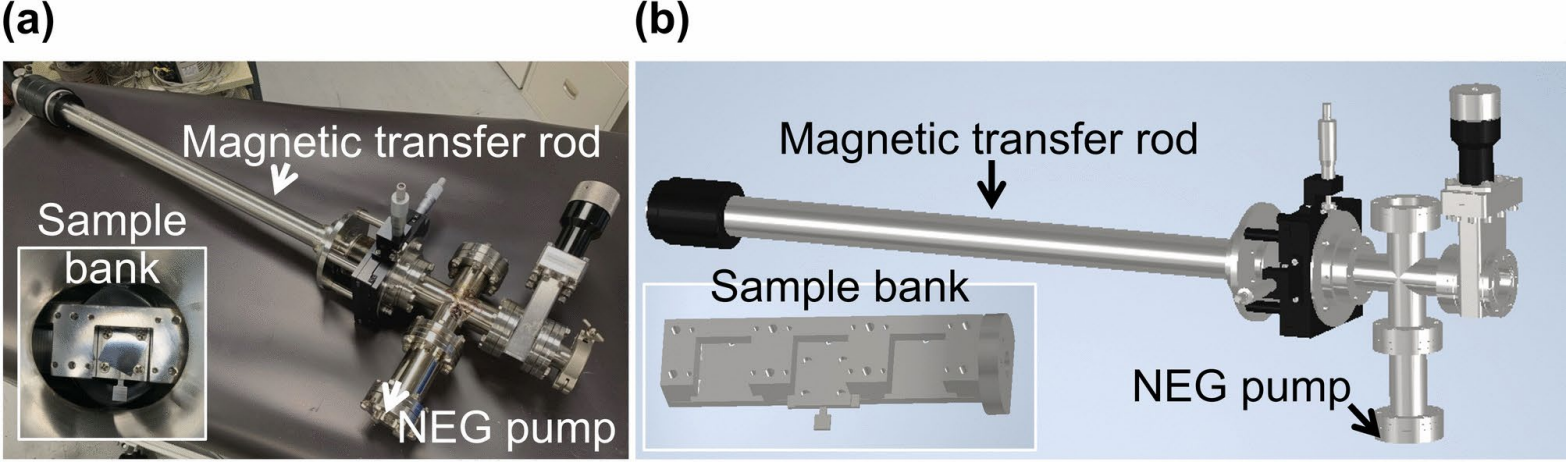
(**a**) photograph and (**b**) schematic of the vacuum suitcase used to transport alkali antimonide photocathodes from the deposition chamber to a synchrotron facility for elemental depth profiling. Insets show the sample banks that go inside of the vacuum suitcase. The location for NEG pump is shown with an arrow.

The vacuum suitcase is shown in Fig. 1. The design is based on a ConFlat 2.75-inch system and used a magnetic transfer rod to minimize outgassing during sample transfer, and a non-evaporable getter (NEG) pump with 200 L/s pumping capability. The total weight is 10 kg, making it light enough for one person to carry.

After baking the system for a week, we first connected the suitcase to our deposition chamber and placed the potassium cesium antimonide samples inside. The pressure inside the vacuum suitcase was monitored throughout, and by ensuring a base pressure of 10^–8^ Pa, we successfully transported our photocathodes from Nagoya University to a synchrotron facility approximately 15 km away (the Aichi Synchrotron Radiation Center)^32^. Next, we transferred the samples to the synchrotron radiation x-ray photoelectron spectroscopy (SR-XPS) analysis chamber.

**Fig. 2:**
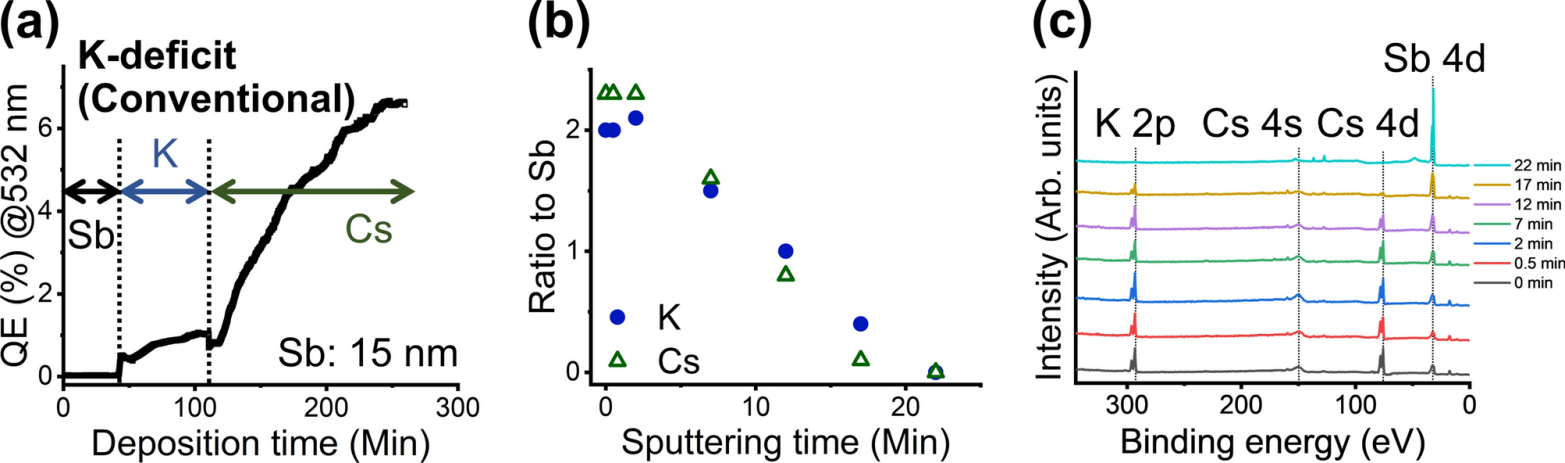
(**a**) The QE of alkali antimonide photocathode thin films with 532 nm excitation during deposition. The films were deposited using our conventional K-deficit recipe of sequential deposition via thermal evaporation. Deposition time is in minutes and vertical dashed lines indicate when a deposition element was changed. (**b**) Depth profile of the deposited film measured using SR-XPS. Sputtering time is in minutes and the filled blue circles indicate the stoichiometric ratio of K to Sb; the open green triangles indicate the stoichiometric ratio of Cs to Sb. (**c**) SR-XPS spectra used to obtain depth profile. The black dotted lines are eye guides for peak positions of each element. The data labels are sputtering times in minutes.

Our initial interest was to obtain depth profiles for potassium cesium antimonide thin films grown with our conventional recipe of sequential deposition via thermal evaporation. We used graphene-coated silicon or molybdenum for our study after confirming that the QEs of deposited photocathodes are identical (i.e., 6.3–6.6% at 532 nm). This is consistent with our previous report, which demonstrated that silicon and molybdenum behave the same regarding the quantum efficiency of deposited K_2_CsSb when they are coated with graphene^33^. The QE of the films with 532 nm excitation during deposition is shown in Fig. 2** (a)**. We monitored the QE using a metal ring anode and a picoammeter. Our deposition recipe is as follows. First, we thermally clean the substrates by heating to around 500 °C for 1 h. After cleaning, we decrease the temperatures to 100 °C and maintain it throughout the deposition. We deposit approximately 15 nm of antimony, which does not yield a detectable increase in the QE. Next, we deposit potassium and start to see some increase in the QE (in the range of 1%). We stop potassium depositions when the QE reaches its peak. Finally, we finish with cesium deposition, during which we see a gradual increase of QE up to approximately 7%. We transported the photocathodes for SR-XPS depth profiling using our vacuum suitcase. The stoichiometry ratio of potassium (K) and cesium (Cs) to antimony (Sb) was approximately two for as-grown films (i.e., prior to sputtering). The results indicate that the films have excess cesium at the surface with respect to the theoretical stoichiometry of crystalline potassium cesium antimonide K_2_CsSb. We cannot conclude whether excess cesium is forming a cesium monolayer on the surface or is incorporated in the films. Regardless, the results provide the important insight that using our described recipe, excess cesium exists on the surface of deposited potassium cesium antimonide.

It is common knowledge that cesium termination of alkali antimonide films leads to a higher QE, and we deposit cesium as the last step in our recipe. Therefore, it is not a surprise that our films possess excess cesium at the surface. What was surprising, however, was the results of depth profiling. We observed an immediate decrease in the ratio of potassium and cesium to antimony with respect to film depth (Fig. 2** (b)**). The rate of decrease was nearly linear with distance and was constant throughout the entire film thickness.

**Fig. 3:**
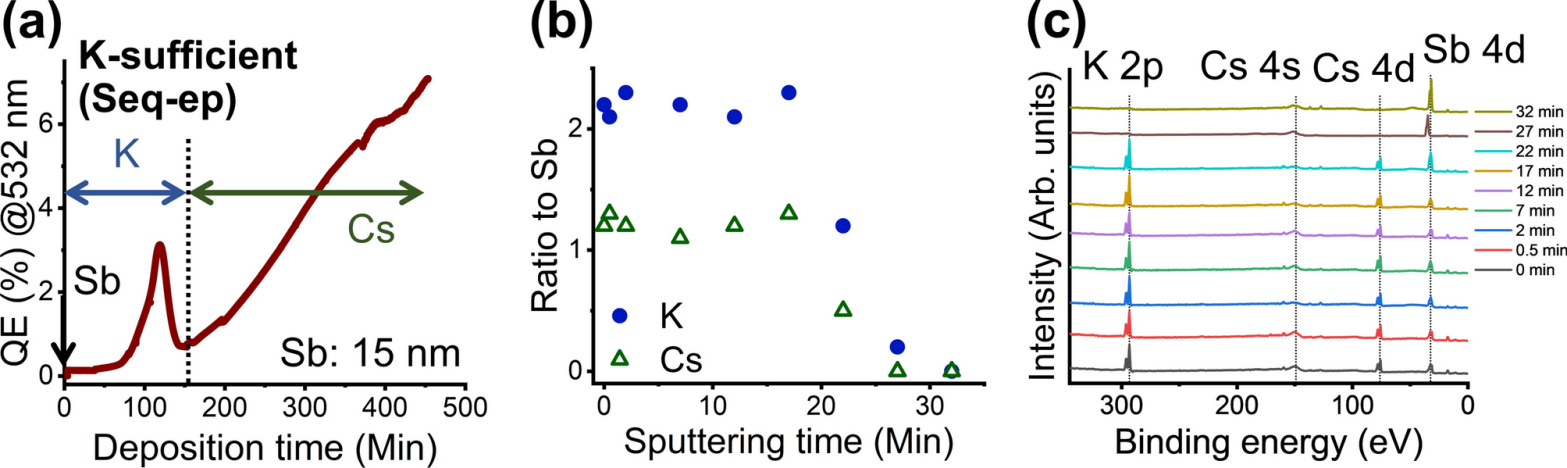
(**a**) The QE of alkali antimonide photocathode thin films with 532 nm excitation during deposition. The films were deposited using the excess potassium recipe of sequential deposition via thermal evaporation (Seq-ep method). Deposition time is in minutes, and the vertical dashed line indicates when a deposition element was changed. (**b**) Depth profile of the deposited film measured using SR-XPS. Sputtering time is in minutes and the filled blue circles indicate the stoichiometric ratio of K to Sb; open green triangles indicate the stoichiometric ratio of Cs to Sb. (**c**) SR-XPS spectra used to obtain depth profile. The black dotted lines are eye guides for peak positions of each element. The data labels are sputtering times in minutes.

After determining that our films did not possess the stoichiometry of crystalline K_2_CsSb, we increased the potassium content during deposition in an attempt to achieve that stoichiometry (using the sequential excess potassium [Seq-ep] method). We hypothesized that the excess cesium at the surface of our films is due to a potassium deficit for cesium to diffuse into the film. Fig. 3** (a)** shows the QE to deposition time plot for the Seq-ep method. Specifically, we waited until the QE dropped off completely rather than stopping at the peak during potassium deposition. All other procedures were maintained. With this adjustment, we did not observe much change in the maximum QE of the films, which was approximately 7% with 532 nm excitation. We transported the films for SR-XPS using the vacuum suitcase for further investigation via depth profiling. Fig. 3** (b)** shows the stoichiometry ratio of K and Cs to Sb.

 Interestingly, we observed an immediate difference in surface elemental composition: It was close to K_2_CsSb and without excess cesium. Even more interesting was that the stoichiometry of K_2_CsSb was maintained considerably deeper into the films, in contrast to the films deposited using our conventional K-deficit method. These results suggest that increasing the potassium content in sequential deposition of potassium cesium antimonide could have supplied sufficient potassium for cesium to react to form the theoretical stoichiometry of K_2_CsSb. However, obtaining the same maximum QE for films with a clear difference in surface stoichiometries and depth profiles emphasizes the need for caution in assuming the stoichiometry of alkali antimonide photocathode thin films based on their QE. Our results indicate that this is especially true for sequentially deposited alkali antimonide thin films. Our depth profiles suggest cesium diffusion is enhanced for the K sufficient films compared the K deficit films. A possible explanation is that there is a difference in the average density of atomic packing for K-Sb films deposited by the two methods. Specifically, an in situ x-ray diffraction study on sequential thermal deposition of K-Cs-Sb films has revealed that cubic K_3_Sb coexists with hexagonal K_3_Sb prior to the cesium deposition^20^. K-Sb in our K-sufficient films may have lower average density atomic packing structure, for example, with increased cubic K_3_Sb crystal structure compared to hexagonal K_3_Sb.

Following stoichiometric evaluations, we proceeded with photocathode robustness tests against corrosive gases. We used different samples made under the same procedures for robustness tests and XPS depth profiling. QE for all samples was 6.3–6.6% at 532 nm. Specifically, we performed pressure-dependent measurements of QE with 532 nm excitation under oxygen gas. 

**Fig. 4:**
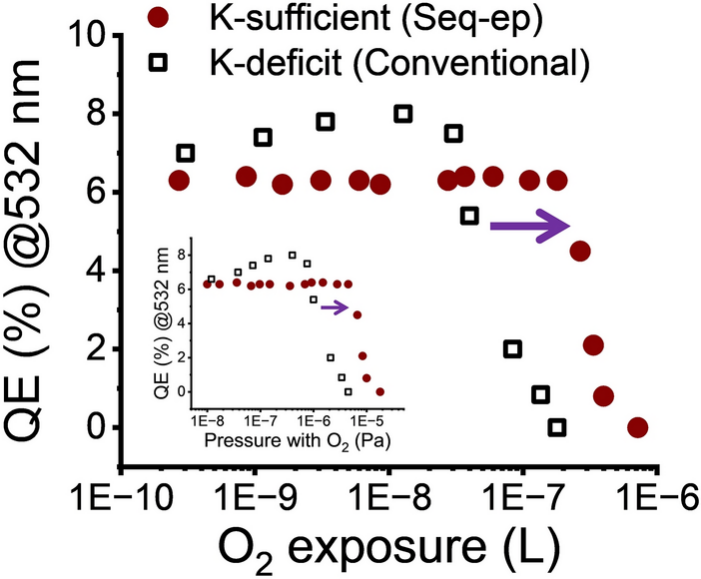
Oxygen exposure-dependent measurement results of QE with 532 nm excitation for alkali antimonide photocathodes deposited using our Seq-ep (filled red circles) and conventional (open black squares) methods. The exposure is in Langmuir. The inset shows the exposure in pressure (Pa). Purple arrows are for eye guide to show the improved robustness for K-sufficient photocathode (i.e., deposited using the Seq-ep method).

The results are shown in Fig. 4 in oxygen exposure and pressure. Unexpectedly, films deposited using our Seq-ep method did not exhibit a QE decrease, even at one order of magnitude higher pressure, compared to those deposited using the conventional K-deficit method. QE remain unchanged until the pressure reached 4.5 × 10^–6^ Pa for films deposited using the Seq-ep method and until 4.0 × 10^–7^ Pa for films deposited using the conventional K-deficit method. These results again highlight the importance of synthesizing potassium cesium antimonide with high enough potassium content to obtain the theoretical stoichiometry of crystalline K_2_CsSb for achieving both high QE and improved robustness. The slight QE increase for films deposited using the conventional K-deficit method as pressure increases from 1.2 × 10^–8^ Pa to 4.0 × 10^–7^ Pa is another distinct difference between K-deficit and K-sufficient films. The QE increase is consistent with a previous report^34^ and could be due to surface dipole formation from oxidation of the cesium surface layer as is the case for GaAs. Cesiating GaAs surfaces and exposing them to oxygen to form negative-affinity surfaces as electron sources for accelerators is an established technique. This could imply that excess cesium on our potassium cesium antimonide deposited using the conventional K-deficit method is at the film surface.

Density functional theory (DFT) calculations were performed to gain insight into the origin of robustness difference of photocathodes with different compositions. The surfaces of K_2_CsSb were represented in a three-dimensional periodic-boundary condition simulation with a vacuum gap in the direction perpendicular to the surface. In the supercell, the vacuum distance normal to the slab was larger than 20 Å to eliminate the interactions between the replicas due to the periodic boundary conditions in the direction normal to the surface. The low index (111) surfaces of K_2_CsSb were considered. The adsorption energy of oxygen molecules on the surface is defined as.

E_ads_ = E_total_ – (E_slab_ + E_O2_),

where E_total_ is total energy after the adsorption of O_2_ on K_2_CsSb, and E_slab_ is the total energy of the slab. A negative value of E_ads_ corresponds to an exothermic process, which means oxygen molecules will spontaneously adsorb on the surface^35^.

**Fig. 5:**
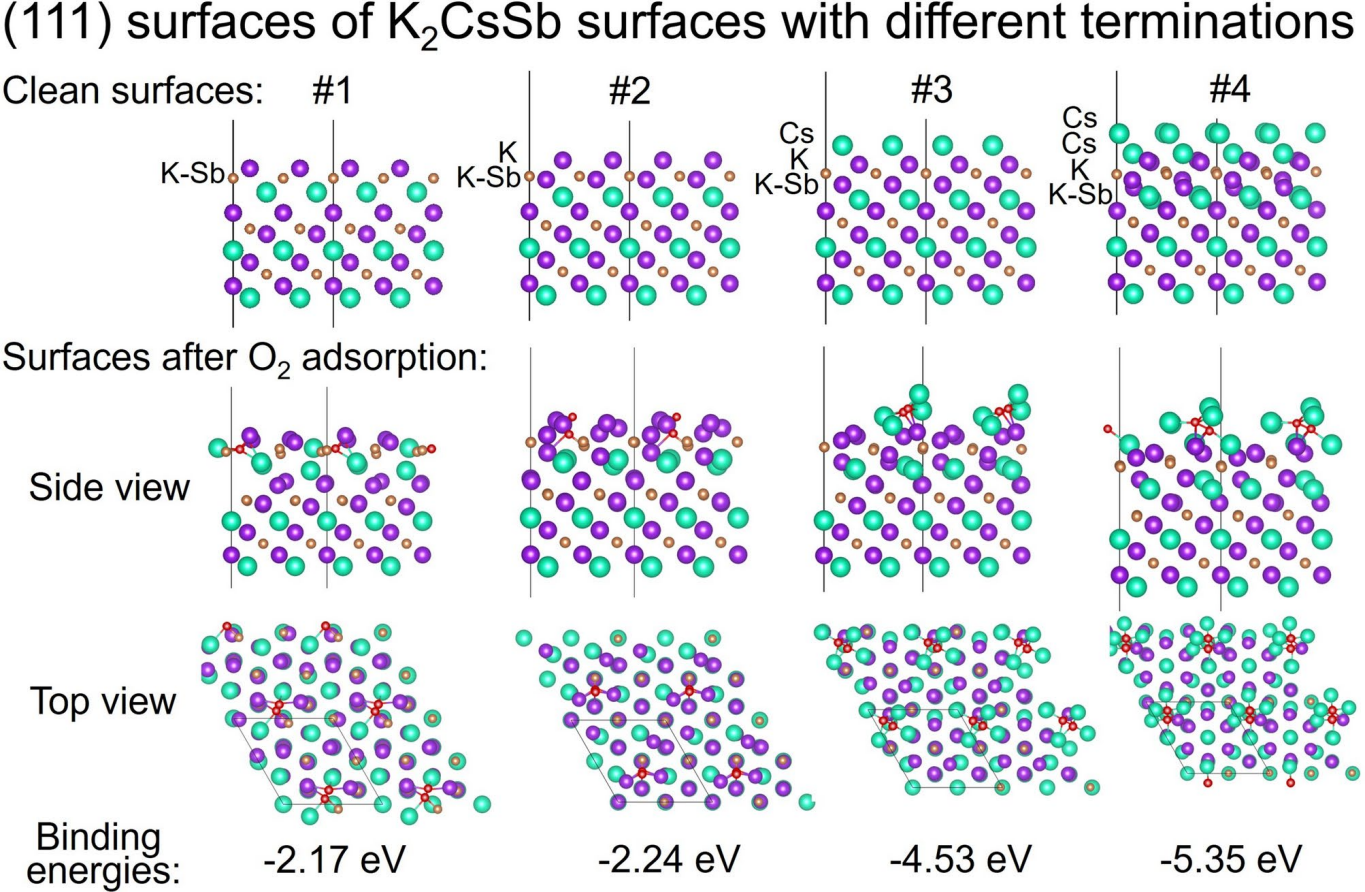
**(Top half)** Structures of clean surface of K_2_CsSb (111) and the surfaces with different terminations used for DFT calculations. Each surface is labeled surface 1–4. **(Bottom half)** The clean surfaces after O_2_ adsorption. There are side view images (top) and top view images. Purple circles indicate potassium, brown circles indicate antimony, green circles indicate cesium, and red circles indicate oxygen.

Figure 5 shows the structures of clean surfaces of K_2_CsSb and the surfaces after O_2_ adsorption. Surfaces of potassium cesium antimonide with different potassium and cesium terminations and contents were considered: clean (111) surface terminated with one K layer (surface 1), surface terminated with two K layers (surface 2), surface terminated with one layer of Cs (surface 3), and finally surface 3 with an additional Cs layer on top (surface 4). Oxygen molecules were adsorbed onto each surface, and the corresponding binding energies were calculated using the described equation. We obtained -2.17, -2.24, -4.53, and -5.35 eV for these surfaces, respectively. The large negative binding energies indicate that oxygen molecules chemically react with these surfaces. The binding strengths of oxygen molecules on Cs-terminated surfaces (-4.53 eV for surface 3 and -5.35 eV for surface 4) are clearly stronger than those on K-terminated surfaces (-2.17 eV for surface 1 and -2.24 eV for surface 2). This implies that oxygen molecules have much stronger chemical reactivity with Cs-terminated surfaces than K-terminated surfaces such that oxidation progresses rapidly to cause the QE decrease. These results are consistent with our experimental results, according to which photocathodes with Cs termination and higher Cs content on the surface have less robustness towards oxygen compared to those with crystalline stoichiometry. The progression of oxidation beyond the surfaces is expected to be via structural defects and grain boundaries.

**Fig. 6:**
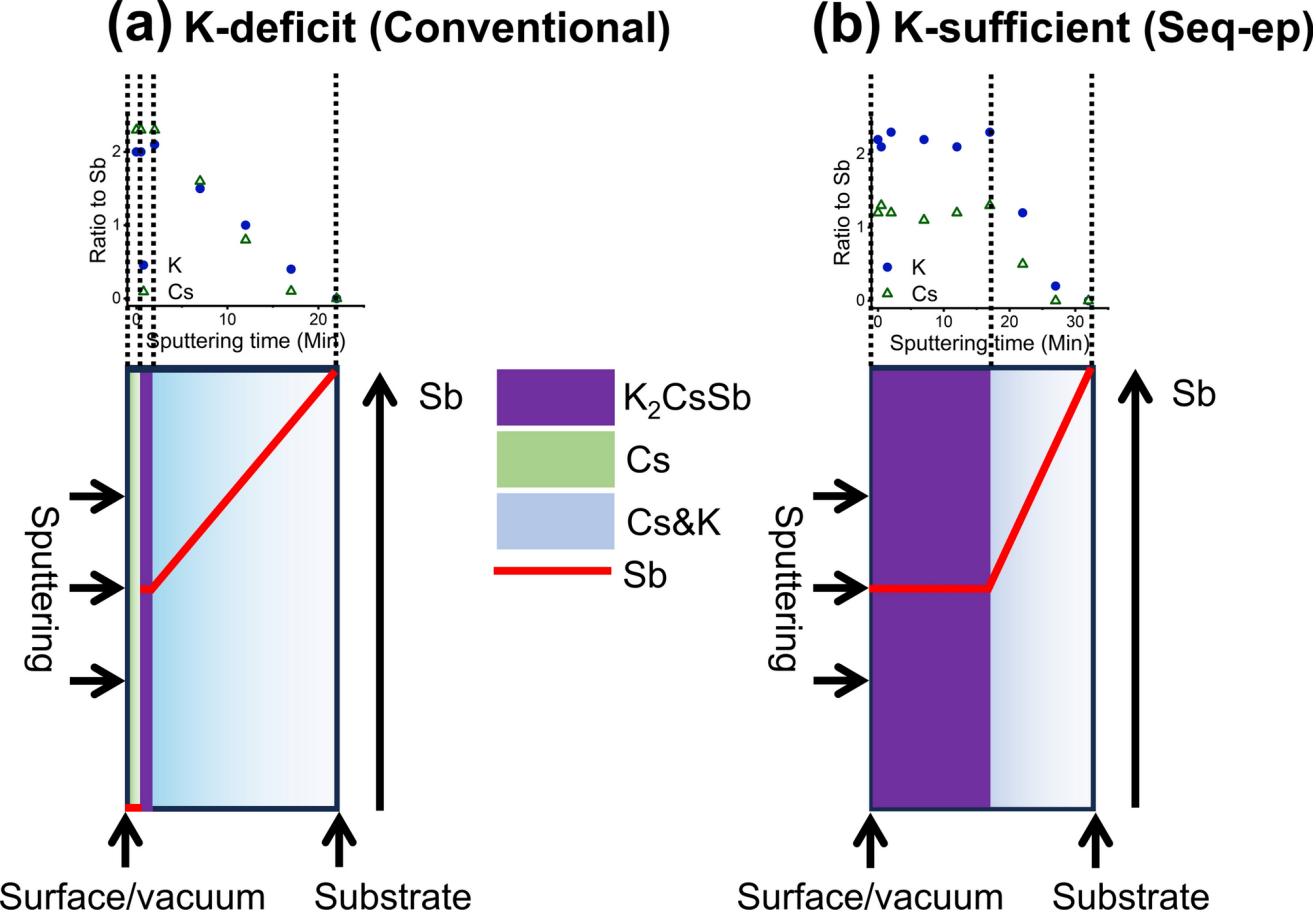
Illustration of the different elemental compositions of alkali antimonide photocathode thin films revealed by SR-XPS depth profiling. **(a)** K-deficit thin film deposited by our original recipe. **(b)** K-sufficient thin film deposited by the modified recipe. The left side is surface/vacuum where films were sputtered, and the right side is substrate. The purple region represents K_2_CsSb, green region represents Cs, and blue region represents Cs&K with gradation indicating decrease in its stoichiometric ratio to Sb. The amount of Sb is indicated by the red line with higher content towards top. The graph on top for (a) is Fig. 2 (b) and the graph for (b) is Fig. 3 (b).

A summary of the results of this study is presented in Fig. 6, which illustrates the different elemental compositions of alkali antimonide photocathode thin films revealed by SR-XPS depth profiling. Specifically, films deposited using the conventional K-deficit method that stops potassium deposition at the QE peak have excess cesium at the surface and an immediate decrease in the ratio of cesium and potassium to antimony with depth. The rate of decrease was linear with respect to the distance throughout the entire film. Therefore, these films only possessed a very thin layer of crystalline K_2_CsSb stoichiometry beneath the excess cesium layer at the surface, if any. However, films deposited using our Seq-ep method, which stops potassium deposition only after the QE drops off completely have crystalline K_2_CsSb stoichiometry that persists throughout half of the entire film thickness. After that, the ratio of cesium and potassium to antimony decreases linearly, similar to films deposited using the conventional K-deficit method.

## Methods

### Substrates for K_2_CsSb photocathodes

Graphene-coated molybdenum substrate was used for XPS depth profiling of K-deficit K_2_CsSb photocathode (deposited by conventional method). Graphene-coated silicon substrates were used for XPS depth profiling of K-sufficient K_2_CsSb photocathodes (deposited by the Seq-ep method) and oxygen exposure of both K-deficit and K-sufficient K_2_CsSb photocathodes. QE for all samples was 6.3–6.6% at 532 nm.

### SR-XPS measurements and depth profiling

SR-XPS measurements were performed using beamline BL7U of the Aichi Synchrotron Radiation Center. All SR-XPS spectra were measured with 400 eV of the x-ray incident energy, thus, the analysis depth is approximately 1 nm from the surface. We used an argon ion gun at 3 keV to sputter the sample surface and performed SR-XPS after different accumulated sputtering times for depth profiling.

### SR-XPS data analysis

SR-XPS data analysis was performed using CasaXPS software. After subtraction of Shirley background, the K 2p, Cs 4d and Sb 4d peak areas were used to determine elemental compositions, taking their cross-sections into account. The stoichiometric ratio of K and Cs was normalized to that of Sb.

### Photocathode robustness tests

Photocathode robustness tests were performed using a gas induction chamber connected to an alkali antimonide thin film deposition chamber. The deposition chamber and robustness test chamber are separated by a gate valve. The system is equipped with a transfer rod capable of transferring photocathodes between the two chambers without exposure to air. A variable leak valve on the gas induction chamber controls gas leak to maintain 10^–8^ Pa. Oxygen gas is connected to the leak valve, and the QE of photocathodes is monitored using a 532 nm excitation source, a metal ring anode, and a picoammeter as the gas is induced into the gas induction chamber. We used different samples made under the same procedures for robustness tests and XPS depth profiling.

### DFT calculations

Calculations were performed using a projector augmented wave (PAW) method as implemented in the Vienna ab initio simulation package (VASP)^36,37^. Plane wave basis sets with a cutoff energy of 500 eV were used. The generalized gradient approximation (GGA) of the Perdew-Burke-Ernzerhof (PBE) functional was used to represent the exchange–correlation interaction^38^. The Sb 5s5p, Cs 5s5p6s, K 3p4s, and O 2s2p electrons were treated as valence electrons. The energy convergence was set to 10^−5^ eV, and the residual force on each atom was smaller than 0.01 eV/Å for structural relaxations.

## Data availability statement

The datasets generated and analyzed during the current study are available from the corresponding author on reasonable request.
